# Characterization of Trileucine Motif in the C-Terminus of the Equine Lutropin/Choriogonadotropin Receptor

**DOI:** 10.3390/cimb46110786

**Published:** 2024-11-18

**Authors:** Sang-Gwon Kim, Munkhzaya Byambaragchaa, Sei Hyen Park, Ha-Rin Jeong, Jae-Hyek Park, Myung-Hum Park, Myung-Hwa Kang, Kwan-Sik Min

**Affiliations:** 1 Graduate School of Animal Biosciences, Hankyong National University, Anseong 17579, Republic of Korea; tom0391@naver.com (S.-G.K.); mrtree119@naver.com (S.H.P.); 2 Carbon-Neutral Resources Research Center, Institute of Genetic Engineering, Hankyong National University, Anseong 17579, Republic of Korea; munkhzaya_b@yahoo.com; 3 Division of Animal BioScience, School of Animal Life Convergence Sciences, Hankyong National University, Anseong 17579, Republic of Korea; jhlin27@naver.com (H.-R.J.); pjh624@naver.com (J.-H.P.); 4 TNT Research, Sejong 30141, Republic of Korea; pmh@tntresearch.co.kr; 5 Department of Food Science and Nutrition, Hoseo University, Asan 31499, Republic of Korea; mhkang@hoseo.edu

**Keywords:** equine LH/CGR, cAMP response, loss of cell surface receptors, pERK1/2

## Abstract

The lutropin/chorionic gonadotropin receptor (LH/CGR) belongs to the G protein-coupled receptor family, characterized by conserved leucine residues in their carboxyl-terminal cytoplasmic tails. This study aimed to investigate the functional significance of the equine LH/CGR (eLH/CGR) trileucine motif in signal transduction. Wild-type eLH/CGR (eLH/CGR-wt) and mutant receptors, in which the trileucine motif was altered to alanine (eLH/CGR-ALL, LAL, LLA, and AAA), were analyzed in transfected cells. The expression levels of mutants ranged from 60% to 78%, with eLH/CGR-AAA showing the lowest level. Although the trileucine motif did not individually affect cAMP responsiveness, the combined mutant (eLH/CGR-AAA) significantly reduced cAMP response, surface receptor levels and enhanced receptor internalization rates. Activation of phospho-ERK1/2 was rapid in all mutants, peaking at 5 min, but eLH/CGR-ALL and LAL mutants exhibited a sharp decline in activity at 15 min. Notably, the eLH/CGR-LLA and AAA mutants showed similar phospho-ERK1/2 activity as the wild type. The eLH/CGR-AAA mutant also displayed a two-fold reduction in PKA signal transduction. These findings suggest that while individual leucine residues of the trileucine motif do not affect cAMP responsiveness, the entire motif plays a crucial role in receptor trafficking and signaling, specifically influencing PKA and phospho-ERK1/2 pathways.

## 1. Introduction

G protein-coupled receptors (GPCRs) constitute the largest class of transmembrane cell surface receptors, regulating various intracellular signaling pathways in response to a wide range of extracellular stimuli [1,2]. The lutropin/choriogonadotropin receptors (LH/CGRs) are members of this GPCR family [3]. These receptors are well-characterized within the broader group of seven transmembrane receptors (7TMRs), which mediate intracellular effects through G protein activation [4,5]. LH/CGRs play a pivotal role in receptor-mediated responses such as cyclic AMP (cAMP) production [6,7] and the activation of extracellular signal-regulated kinases (pERK1/2) [8,9,10].

Receptor-mediated endocytosis of LH/CGRs serves two critical functions in modulating cellular responsiveness. First, the internalization of the LH/CGR-bound agonist inhibits the activation of downstream receptors involved in intracellular signaling [11]. Second, the persistent formation of the agonist–receptor complex within lysosomes promotes receptor degradation and agonist-induced downregulation of cell surface LH/CGRs [12,13]. This internalization process is key for GPCR recycling back to the plasma membrane, which plays a crucial role in receptor resensitization [14].

Agonist stimulation of GPCRs triggers Gs-mediated activation of adenylyl cyclase, leading to increased cAMP production and the subsequent activation of downstream signaling pathways [15,16]. Both Gαs proteins and β-arrestins are involved in ERK signaling via two temporally distinct mechanisms: a G protein-dependent mechanism that is rapid in onset and a slower β-arrestin-dependent mechanism [16,17]. β-arrestins, particularly β-arrestin 1 and 2, are implicated in pERK1/2 activation in LH/CGRs and follicle-stimulating hormone receptors (FSHRs) [13,18,19,20]. Notably, phospho-ERK1/2 activation in both LH/CGR and FSHR occurs rapidly, within 5 min of agonist stimulation [21,22,23]. In human LH/CGR (hLH/CGR), pERK1/2 phosphorylation was similarly enhanced within 5 min following treatment with hCG agonist, suggesting that this effect is mediated through a cAMP/PKA-dependent pathway [13,24].

Equine LH/CGR (eLH/CGR) binds both equine chorionic gonadotropin (eCG) and luteinizing hormone (eLH). eLH is a glycoprotein hormone secreted by the pituitary gland, while eCG, also a glycoprotein, is secreted by trophoblastic cells into the bloodstream of pregnant mares between the second and fifth months of pregnancy, peaking at 70–80 days of gestation [12,25]. Although the β-subunits of human chorionic gonadotropin (hCG) and human luteinizing hormone (hLH) are encoded by different genes, a single gene encodes both the β-subunit of CG and LH in horses [26,27,28].

**Figure 1:**
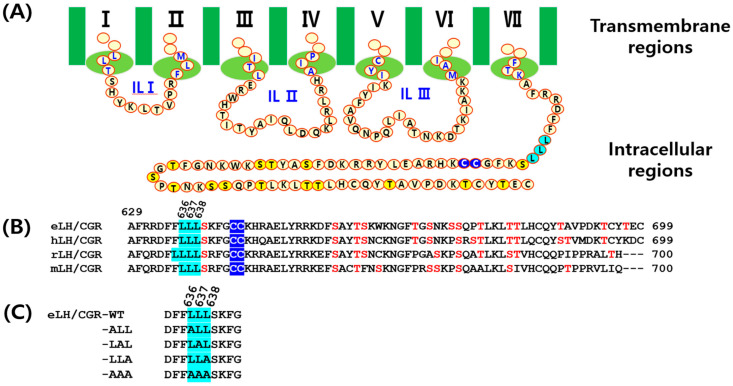
Schematic representation of the structure of equine LH/CGR-wt and mutants. (**A**) The amino acid sequences of the intracellular loops I–III and C-terminal region of eLH/CGR are shown. (**B**) A comparison of the amino acid sequences between eLH/CGR and mammalian LH/CGRs in the C-terminal intracellular regions is provided. (**C**) The trileucine motif mutants of the C-terminus are displayed. Numbers indicate the amino acid positions of the mature protein, excluding the signal sequence. The trileucine region, commonly found in the C-terminus of mammalian LH/CGRs, is highlighted in sky blue. Notably, rLH/CGR contains four leucine residues in this region. Potential phosphorylation sites are marked yellow and red in panels (**A**,**B**), while palmitoylation sites at intracellular cysteine residues are indicated in blue in panel B.

Most GPCRs contain multiple sites for downstream signaling in their C-terminal regions, including phosphorylation sites (serine and threonine residues), conserved palmitoylation sites (two cysteines), and a trileucine motif. The conserved leucine sequence functions as a binding site for adaptor proteins necessary for intracellular protein trafficking in rat LH/CGR (rLH/CGR) and hLH/CGR [29]. The deleucine-based motifs in rLH/CGR inhibit internalization for impairment in the binding of the rLH/CGR to the endogenous nonvisual arrestins. Among them, several LH/CGRs have been shown to have a leucine motif in their C-terminal tail as shown in Figure 1.

This trileucine motif is also found in the C-terminus of eLH/CGRs, and in the present study, we investigated the role of these three leucine residues using site-directed mutagenesis. These studies were specifically designed to determine whether trilecuine motif affects the functional properties of the eLH/CGR. Our findings revealed that substituting alanine for Leu636Leu637Leu638 did not completely impair cAMP responsiveness, cell surface expression, or phospho-ERK1/2 activation.

## 2. Materials and Methods

### 2.1. Materials

Oligonucleotides were synthesized, and mutated DNAs were sequenced by Genotech (Daejeon, Republic of Korea). The pGEM-T Easy Cloning Vector was purchased from Promega (Madison, WI, USA). DNA ligation reagents, endonucleases, polymerase chain reaction (PCR) reagents, and restriction enzymes were purchased from Takara Bio (Shiga, Japan). Mammalian expression vector pCORON1000 SP VSV-G was purchased from Amersham Biosciences (Piscataway, NJ, USA). The CHO-K1 cells and HEK 293 cells were obtained from the Korean Cell Line Bank (KCLB, Seoul, Republic of Korea). FreeStyle MAX reagent and Lipofectamine-2000 were purchased from Invitrogen (Carlsbad, CA, USA). Fetal bovine serum (FBS), Ham’s F-12 medium, and Opti-MEM were purchased from Gibco (Grand Island, NY, USA). The cAMP homogeneous time-resolved fluorescence (HTRF) assay kit was purchased from Cisbio (Codolet, France). The Lumi-Light^plus^ Western blotting substrate was obtained from Roche Inc. (Pleasanton, CA, USA). SuperSignal^TM^ West Pico PLUS Chemiluminescent substrate was purchased from Thermo Fisher Scientific (Waltham, MA, USA). Rabbit anti-VSV-G tag monoclonal and polyclonal antibodies, goat anti-rabbit IgG-HRP secondary antibody, pERK1/2 antibodies, total ERK1/2 antibody, and goat anti-mouse HRP-conjugated secondary antibody was purchased from Cell Signaling Technology (Danvers, MA, USA). The QIAprep-Spin plasmid kit was purchased from Qiagen Inc. (Hilden, Germany) and disposable flasks were purchased from Corning Inc. (Corning, NY, USA). CentriPlus centrifugal filters were purchased from Amicon Bio Separation (Billerica, MA, USA). All other reagents were purchased from Sigma-Aldrich (St. Louis, MO, USA).

### 2.2. Site-Directed Mutagenesis and Vector Construction of Equine LH/CGR Trileucine Motif

To construct point mutations in trileucine of the C-terminus for equine LH/CGR, we templated cDNA encoding the full-length eLH/CGR-wild type (eLH/CGR-wt) using an overlap extension PCR strategy to substitute for the alanine (A) amino acid, as described previously [30]. The purified PCR products were subcloned into the pGEMT-Easy vector, and the sequence was confirmed by DNA sequencing. Full-length fragments were cleaved using the XhoI and EcoRI restriction enzymes and ligated into the pCORON SP VSVG mammalian expression vector. The fragments cut by restriction enzymes were eliminated from the signal sequence and inserted under the VSVG tag of the pCORON SP VSVG vector. Finally, the direction of insertion was confirmed by restriction enzyme digestion. Figure 1 shows a schematic diagram of equine LH/CGR-wt and the mutants, denoting the amino acids of the C terminus region in equine LH/CGR.

### 2.3. Transient Transfection into CHO-K1 and HEK 293 Cells

Mammalian expression vectors were transfected into CHO-K1 cells by liposome-mediated transfection. Cells were cultivated in growth medium [Ham’s F-12 media supplemented with penicillin (50 U/mL), streptomycin (50 ug/mL), glutamine (2 mM), and 10% fetal bovine serum]. The HEK 293 cells were cultured in growth medium [Dulbecco’s modified Eagle’s medium containing Hepes (10 mM), gentamycin (50 ug/mL), and 10% fetal bovine serum].

The cells were grown to 80–90% confluence in 6-well plates. Each plasmid DNA and transfection reagent were mixed with Opti-MEM and incubated for 20 min at room temperature. The cells were washed with Opti-MEM and the DNA-Lipofectamine complex was added. After 5 h, fresh growth medium containing 20% fetal bovine serum was added to each well. CHO cells were used for cAMP analysis at 48 h post-transfection. The HEK 293 cells were used to investigate the expression of the receptors, cell surface loss of the receptors, and phosphor-ERK1/2 analysis.

### 2.4. Quantitation of Equine LH/CGR Expression Using Western Blotting

The transfected HEK 293 cells were harvested and solubilized in RIPA buffer (50 mM Tris-HCl, pH 8.0, 150 mM NaCl, 0.1% SDS, 1% Triton X-100, and 0.5% sodium deoxycholate) with a protease inhibitor cocktail. For Western blot analysis, the collected proteins (20 µg) were subjected to 10% sodium dodecyl sulfate poly-acrylamide gel electrophoresis (SDS-PAGE). Proteins were transferred onto a polyvinylidene difluoride membrane for 90 min in a mini transblot electrophoresis cell. The membrane was blocked with 5% skim milk and then incubated overnight with the primary antibody, rabbit anti-VSV-G tag monoclonal antibody, diluted 1:1000 times with a blocking solution. After washing three times with TBS-T, the membrane was incubated with goat anti-rabbit IgG-HRP secondary antibody diluted 1:3000 in blocking buffer for 1 h, followed by washing three times with TBS-T. The signal was detected using an enhanced chemiluminescence method with SuperSignal and visualized on an X-ray film.

### 2.5. Analysis of cAMP Levels via Homogenous Time-Resolved Fluorescence Assays

cAMP accumulation in CHO-K1 cells expressing equine LH/CGR-wt or mutants was measured using a cAMP Dynamics 2 competitive immunoassay kit (Cisbio Bioassays, Codolet, France) as previously described [30]. Briefly, transfected cells were diluted in 0.5 mM IBMX to inhibit cAMP degradation and seeded in 384-well plates (10,000 cells per well). And then, rec-eCG ligand (5 μL) was added to each well and incubated for cell stimulation at RT for 30 min. The cryptate-conjugated anti-cAMP monoclonal antibody and d2-labeled cAMP reagent were added to each well, followed by incubation for 1 h at room temperature. cAMP was detected by measuring the decrease in HTRF energy transfer (665 nm/620 nm) using a TriStar2 S LB942 mi microplate reader (BERTHOLD Tech, Wildbad, Germany). The specific signal delta F (energy transfer) was inversely proportional to the concentration of cAMP in the standard or sample. The results were calculated from the ratio of fluorescence at 665 and 620 nm, expressed as Delta F% (cAMP inhibition), according to the following equation: Delta F% = (standard or sample ratio-mock transfection) 100/mock transfection. The cAMP concentration for Delta F% values was calculated using GraphPad Prism software (version 6.0; GraphPad Software Inc., La Jolla, CA, USA).

### 2.6. Agonist-Induced Cell Surface Loss Assay of Equine LH/CG Receptors

The loss of equine LH/CGR at the cell surface was assessed using ELISA as previously described [30]. Cells were plated at a density of 6 × 10^5^ per 60 mm dish and transfected with equine LH/CGR-wt and mutant plasmids. Cells were seeded into 96-well plates (1 × 10^4^ cells) at 24 h post-transfection. The next day, cells were pre-incubated with rec-eCG (250 ng/mL) for time-dependent analyses (0, 5, 15, and 30 min). After washing DPBS, the cells were fixed with 4% paraformaldehyde in DPBS for 5 min. After washing twice with DPBS, the cells were incubated with blocking solution for 30 min, followed by incubation with rabbit anti-VSVG antibody (1:1000) and horseradish peroxidase-conjugated anti-rabbit antibody (1:4000) for 1 h. Cells were washed three times with a blocking solution.

ELISA Femto Maximum substrate (15 uL) and Luminol/Enhancer (15 uL) were added to each well. The luminescent signal was measured using a Cytation 3 plate reader. The expression level of equine LH/CGR-wt was set to 100% at 0 s. The cell surface expression levels of equine LH/CGR-wt and mutants were set to 100% in untreated cells. The expression of cell-surface receptors was calculated by comparing the loss during agonist stimulation to the levels in untreated cells (0% loss of cell-surface receptors).

### 2.7. Phospho-ERK1/2 Time Course

pCORON1000 SP VSV-G plasmids containing equine LH/CGR-wt and mutants were transfected into HEK 293 cells. After 48 h, the cells were starved for at least 4–6 h and then treated with a rec-eCG agonist (250 ng/mL) in a time-dependent manner. Cells were lysed in RIPA buffer (Sigma-Aldrich, St. Louis, MO, USA). Equal amounts (20–40 ug) of cellular extracts were loaded onto 10% SDS-PAGE gels and transferred onto nitrocellulose membranes. Phospho-ERK1/2 and total ERK1/2 were detected by immunoblotting using rabbit polyclonal anti-phospho-p44/42 MAPK (1:2000) and anti-MAPK1/2 (1:3000), respectively. Membranes were then incubated with an anti-rabbit secondary antibody. Chemiluminescence was detected using West Pico reagent, and phosphorylated ERK1/2 immunoblots were quantified by densitometry using ImageLab v. 6.0 (Bio-Rad, Hercules, CA, USA).

### 2.8. Data Analysis

The Multalin multiple sequence alignment tool was used for sequence analysis. Dose–response curves were generated for experiments performed in duplicate. GraphPad Prism 6.0 (San Diego, CA, USA) was used to analyze the cAMP response, EC_50_ values, and stimulation curves. Curves fitted in a single experiment were normalized to background signals measured in mock-transfected cells. Each curve was plotted using data from three independent experiments. The phosphor-ERK1/2 values were calculated using GraFit Version 5 (Erithacus Software, Horley, Surrey, UK). The results are expressed as mean ± standard error of the mean of three independent experiments. Data were analyzed by one-way analysis of variance (ANOVA), followed by Tukey’s comparison test using GraphPad Prism v.6.0, as indicated in the figure captions. Statistical significance was determined at the following levels: * *p* < 0.05 and ** *p* < 0.01, indicating a significant difference between groups.

## 3. Results

### 3.1. Preparation of Equine LH/CGR and Their Cell-Surface Expression

We generated mutants of equine LH/CGR at the tri-leucine motif in the C-terminal region to assess their effects on ligand-receptor interactions (Figure 1). As depicted in Figure 1, multiple conserved phosphorylation, palmitoylation, and trileucine sites were identified. The trileucine motif is located at amino acids 636, 637, and 638 of equine LH/CGR, and these sites are well conserved among LH/CGRs in mammalian species, with the exception of four sites in rat LH/CGR.

**Figure 2:**
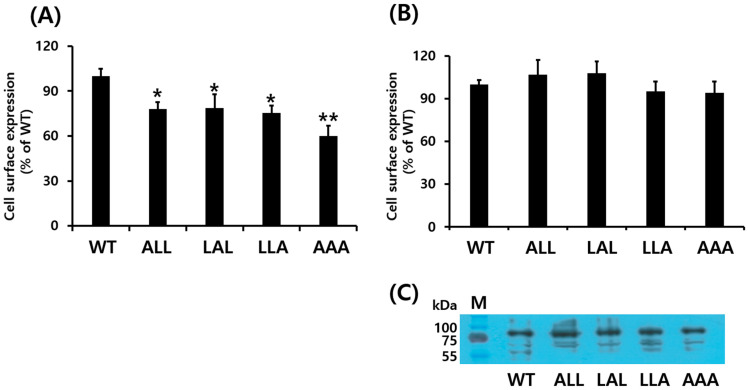
Cell surface expression of eLH/CGRs in transiently transfected HEK 293 cells. (**A**) Enzyme-linked immunosorbent assay (ELISA) and (**B**) Western blotting were used to assess surface expression of eLH/CGRs. Immunoblotting results were quantified via densitometry using Image-Lab software (Bio-Rad). (**C**) Western blots of whole-cell lysates from transfection were probed with antibody to VSVG-tag as shown. Values are expressed as the mean ± standard error of the mean from three independent experiments and normalized to eLH/CGR-wt, which was set to 100%. Statistically significant differences were determined using one-way ANOVA followed by Tukey’s comparison test (* *p* < 0.05, ** *p* < 0.01).

Next, we evaluated the cell surface expression of WT and mutant eLH/CGRs in HEK 293 cells using ELISA. The surface expression of eLH/CGR-wt was set as 100%, with initial measurements at time zero. The expression levels of the eLH/CGR-ALL, LAL, and LLA mutants were approximately 77.9%, 78.7%, and 75.3%, respectively (Figure 2A). The eLH/CGR-AAA mutant exhibited the lowest expression level at 60%. Additionally, receptor protein expression was analyzed via Western blotting (Figure 2B,C), which revealed a major band corresponding to a molecular weight of approximately 80 kDa. Western blot analysis showed higher expression for the eLH/CGR-ALL and LAL mutants compared to WT eLH/CGR, though these results differed slightly from the ELISA data. Nevertheless, the eLH/CGR-AAA mutant consistently showed the lowest expression level across both assays.

### 3.2. Biological Activities of the Equine LH/CGR-wt and Trileucine Mutants

**Figure 3:**
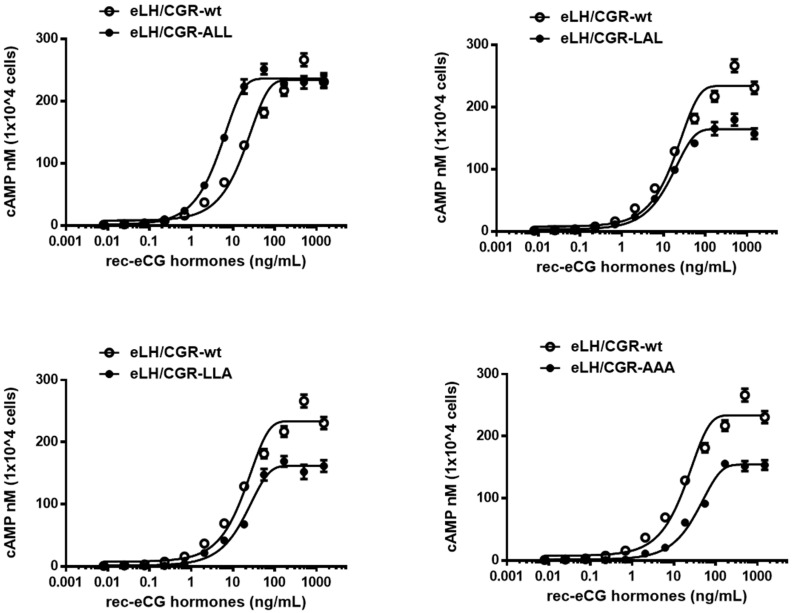
Total cAMP levels in CHO-K1 cells transfected with eLH/CGR-wt and trileucine mutants stimulated by rec-eCG. CHO-K1 cells transiently transfected with eLH/CGRs were seeded in 384-well plates (10,000 cells/well) 48 h post-transfection. The cells were stimulated with rec-eCG for 30 min at room temperature. cAMP production was measured using a homogeneous time-resolved fluorescence (HTRF) assay and expressed as Delta F%. cAMP concentrations were calculated using GraphPad Prism software. Data represent the mean ± standard error of the mean from three independent experiments. Mean data were fitted to a single-phase exponential decay curve.

We assessed the in vitro biological activity of WT and mutant eLH/CGRs in transfected CHO-K1 cells by measuring cAMP activation (Figure 3). The dose–response curve for the eLH/CGR-ALL mutant shifted slightly to the left compared to WT, while the curves for the eLH/CGR-LAL, LLA, and AAA mutants shifted to the right.

eLH/CGR-wt exhibited full biological activity, with an EC_50_ value of 18.5 ng/mL and an Rmax of 233.9 ± 4.9 nM per 10^4^ cells. The biological activity of the eLH/CGR-ALL mutant was higher than that of WT, with an EC_50_ value of 4.6 ng/mL (Table 1), indicating a four-fold increase. However, the Rmax value remained comparable to that of WT (236.1 ± 2.1 nM per 10^4^ cells). The eLH/CGR-LAL and LLA mutants showed biological activities similar to that of WT, but their Rmax values were considerably lower, at 164.4 and 161 nM per 10^4^ cells, representing approximately 70% of WT activity. In the eLH/CGR-AAA mutant, the EC_50_ value for the cAMP response was 35.2 ng/mL, nearly two times lower than that of WT, and the Rmax values were similar to those of the LAL and LLA mutants.

These findings suggest that the eLH/CGR-AAA mutant had the most substantial impact on cAMP responsiveness. While the other mutants (eLH/CGR-LAL and LLA) exhibited reduced Rmax values, they did not affect overall biological activity. The data indicate that the trileucine motif does not independently regulate cAMP responsiveness, but combined mutations within this motif significantly influence biological activity.

### 3.3. Loss of Cell-Surface Receptors After Agonist Treatment

**Figure 4:**
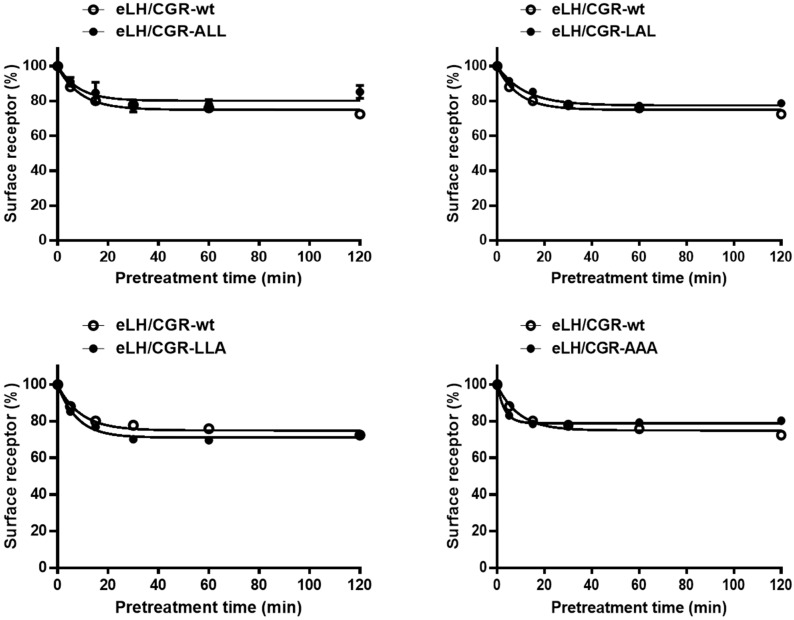
Loss of cell surface expression of eLH/CGR-wt and mutants after agonist treatment. HEK 293 cells transiently transfected with eLH/CGR-wt or mutant plasmids were incubated with or without 250 ng/mL rec-eCG for 120 min. Cell surface receptor expression was then measured and expressed as a percentage of receptor loss compared to initial cell surface levels (set at 100%). Data represents the percentage of receptor loss after agonist pretreatment.

We employed ELISA to evaluate the loss of eLH/CGR from the cell surface following treatment with recombinant eCG (250 µg/mL). HEK 293 cells expressing WT and mutant eLH/CGRs were preincubated with the agonist for 120 min, and the time-dependent reduction in cell surface receptors was analyzed (Figure 4, Table 2). In WT-expressing cells, receptor loss reached approximately 89% within 5 min, decreasing further to 80% at 15 min and 72.7% after 2 h. The surface receptor loss in the eLH/CGR-ALL, LAL, and LLA mutants followed a similar pattern to WT.

In contrast, the eLH/CGR-AAA mutant showed a slight increase in receptor loss, reaching 82% within 5 min, but the loss remained consistent thereafter. The rate of receptor–agonist complex loss from the cell surface was rapid in both WT and mutants, with t1/2 values ranging from 3.1 to 8.0 min (Table 2). For WT eLH/CGR, the t1/2 was 6.3 min, with a maximum reduction of 72.7%. The t1/2 in the eLH/CGR-LAL mutant was slightly delayed, at approximately 8.0 min, while the eLH/CGR-AAA mutant exhibited a notably shorter t1/2 of 3.1 min. These results suggest that individual mutations in the tri-leucine motif do not significantly impact receptor loss from the cell surface. However, the eLH/CGR-AAA mutant accelerated the rate of receptor loss two-fold compared to WT, indicating that receptor loss is not directly linked to cAMP responsiveness in the trileucine motif of eLH/CGR.

### 3.4. pERK1/2 Activation in HEK 293 Cells

**Figure 5:**
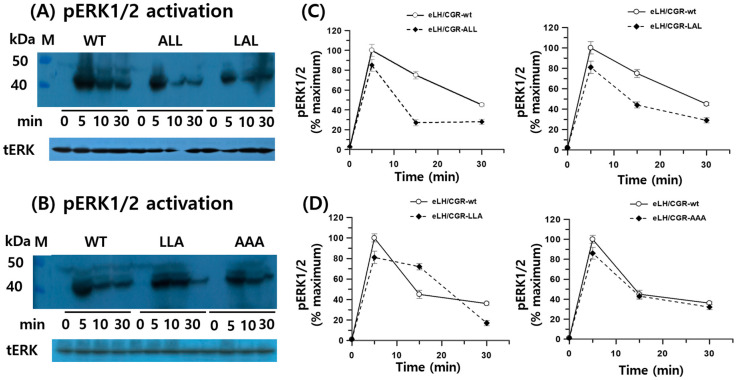
pERK1/2 activation in HEK-293 cells transfected with eLH/CGR-wt and mutants after stimulation with rec-eCG. HEK-293 cells transiently expressing eLH/CGR-wt or mutants were serum-starved for 4–6 h before stimulation with 250 ng/mL rec-eCG for the indicated time periods. Whole cell lysates were analyzed for pERK1/2 and total ERK levels. (**A**,**B**) Western blot results for phospho-ERK1/2 and total ERK. (**C**,**D**) Quantified phosphor-ERK1/2 levels, normalized to total ERK, are expressed as a percentage of the maximal response (100% for eLH/CGR-wt at 5 min). Densitometry was used to quantify the phospho-ERK1/2 band. Representative data are shown, and graphs represent the mean ± SEM of three independent experiments.

In HEK 293 cells transiently expressing WT and mutant eLH/CGRs, recombinant eCG stimulated ERK phosphorylation in a time-dependent manner (Figure 5). In WT cells, rec-eCG induced phospho-ERK1/2 activation within 5 min of agonist treatment, followed by a gradual decline, with 45% of the maximum level remaining after 30 min. Rapid phospho-ERK1/2 activation was also observed in all mutants at 5 min, followed by an abrupt decrease. The phospho-ERK1/2 activity in the eLH/CGR-ALL and LAL mutants dropped to approximately 20% and 40% of the maximum level, respectively, after 15 min of agonist stimulation. No significant differences in phospho-ERK1/2 activity were detected between WT, eLH/CGR-LLA, and eLH/CGR-AAA mutants. These results indicate that the trileucine motif of eLH/CGR plays an independent role in phospho -ERK1/2 activation, receptor surface loss, and PKA signal transduction.

## 4. Discussion

Based on the findings of this study, we conclude that mutations in the trileucine motif within the C-terminus of eLH/CGR do not significantly impact eCG-induced cAMP responsiveness, receptor surface expression, or ERK phosphorylation. However, PKA signaling and Rmax levels were notably decreased in the eLH/CGR-AAA mutant. These results suggest that the conserved leucine motif independently influences downstream signal transduction, though it is not essential for eCG-induced ERK phosphorylation.

As expected from these results, the trileucine mutations led to reduced receptor expression. Specifically, the eLH/CGR-AAA mutant showed a significant decrease in expression according to ELISA analysis, while Western blot data revealed no notable difference, despite the slightly lower expression. Thus, we propose that the trileucine motif in the C-terminus of eLH/CGR does not significantly affect either cell surface expression or the processing of the receptor as an intracellular precursor as shown in Western blot results. However, ELISA’s results are taken to be more accurate than those of the Western blot. Nevertheless, the motif may influence surface expression cooperatively. In previous studies on specific receptor sites, we observed a 50% decrease in cell surface expression of active and inactive LH/CGR mutants, indicating a conformational change [3]. These findings align with previous reports showing that mutations in the dileucine motif (Leu-339 and Leu-340) of the β2-adrenergic receptor had minimal effects on surface expression, ligand binding, G protein coupling, or signaling in CHO and HEK-293 cells [31]. Additionally, studies on rat LH/CGR showed that mutations in dileucine-based motifs inhibited internalization, likely due to impaired binding [29]. We also confirmed that signaling activity in the eLH/CGR-AAA mutant decreased two-fold, indicating that each trileucine residue independently affects signal transduction, which increased cAMP responsiveness in the eLH/CGR-ALL mutant.

Interestingly, our findings suggest a discrepancy in receptor internalization compared to previous studies on rat LH/CGR [29] and the β2-adrenergic receptor [31], both of which reported significant impairments in agonist-induced internalization in mutants. In contrast, our data suggest that the trileucine motif in eLH/CGR does not affect receptor internalization but rather influences the loss of cell surface receptors. Thus, the role of the trileucine motif in eLH/CGR internalization requires further investigation. These leucine motifs are located near accessible phosphorylation sites on adjacent serine/threonine residues and palmitoylated cysteines, contributing to the structure of the receptor’s C-terminal region. Although we did not analyze phosphorylation or palmitoylation directly, previous studies have demonstrated that rat LH/CGR (C621-622) is palmitoylated, and mutations in this region led to intracellular trapping of the receptor, as observed in the δ-opioid receptor [32,33]. Therefore, it is plausible that the leucine motif is closely linked to phosphorylation sites and palmitoylation.

Previous research has suggested that dileucine motifs bind to adaptor protein complexes, such as AP1 and AP2, which are involved in GPCR internalization through clathrin-mediated endocytosis, followed by lysosomal degradation or recycling back to the membrane for sustained agonist stimulation [34,35]. β-arrestins function as receptor/clathrin adaptors, binding to phosphoserine and phosphothreonine residues [36]. The phosphorylation-dependent conformation of the GPCR C-terminal region modulates arrestin binding, thereby affecting arrestin-mediated signaling outcomes [37]. Thus, conformational changes in serine and threonine residues in the GPCR C-terminal domain play a critical role in downstream signaling pathways.

In this study, we found that the rate of receptor loss from the cell surface was not affected by leucine motif mutations, but eLH/CGR-AAA mutants exhibited faster receptor loss compared to eLH/CGR-wt. This suggests that the trileucine motif in eLH/CGR does not directly regulate cell surface receptor loss. Notably, we also observed that receptor loss occurred rapidly despite the complete impairment of the cAMP response [3]. As highlighted in recent studies [38,39], this mechanism remains poorly understood. We hypothesize that the trileucine motif may be involved in a phosphorylation and β-arrestin-dependent pathway contributing to eLH/CGR internalization.

ERK activation is critical for many GPCR-mediated physiological responses [19]. ERK1/2 phosphorylation involves the sequential activation of Raf1, MEK1, and ERK1/2 kinases [40]. LH-induced intracellular signaling triggers Gs-mediated activation of adenylyl cyclase, resulting in cAMP production in a G protein-dependent manner [41]. This ERK1/2-mediated signaling pathway has been observed in many GPCRs that interact with β-arrestins, such as the β2-adrenergic receptor [16], u-opioid receptor [42], glucagon-like peptide-1 receptor [14], and angiotensin II receptor [43]. In this study, we observed that phospho-ERK1/2 levels in eLH/CGR-wt mice peaked at 5 min following agonist stimulation. These results are consistent with previous studies on FSH- and hCG-induced phospho-ERK1/2 activation, which showed a similar peak around 5–6 min [13,19]. The eLH/CGR-ALL and LAL mutants showed similar levels of activation within 5 min, with a decline by 30 min, while the eLH/CGR-LLA and eLH/CGR-AAA mutants did not significantly affect phospho-ERK1/2 activation.

Our findings align with studies on hLH/CGR, where phospho-ERK1/2 activation was relatively minor compared to cAMP, progesterone, and inositol phosphate signaling [44]. In contrast to many GPCRs, β-arrestin-mediated internalization of FSHR was not required for ERK activation by FSH [18,19]. This suggests that the phospho-ERK1/2 response is mediated by G proteins distinct from those involved in cAMP signaling. Our data indicate that both cAMP and phospho-ERK1/2 responses may follow pleiotropic patterns, with ERK activation occurring through a G protein-independent mechanism.

Recent studies on phospho-ERK1/2 activation have highlighted the role of G protein-coupled receptor kinases (GRKs) and β-arrestins, with knockdown and knockout models showing reduced ERK activation that was restored by reintroducing β-arrestin genes [18,20]. These results were observed in GPCRs such as the β2-adrenergic receptor, vasopressin V2 receptor, and u-opioid receptor [20,42]. Future studies should focus on elucidating the mechanism of phospho-ERK1/2 activation in β-arrestin 1 or 2 knockout models to better understand LH/CGR signaling pathways. Based on our results, phospho-ERK1/2 in eLH/CGR-wt cells was activated in a dose-dependent manner, similar to other GPCRs. The trileucine motif in the C-terminus of eLH/CGR is not essential for cell surface expression, receptor loss, or phospho-ERK1/2 activation.

The results clearly demonstrate that each leucine residue had no effect on the properties of eLH/CGR including PKA signal transduction, loss of cell surface receptor, and phospho-ERK1/2. eLH/CGR-AAA mutant, however, specifically influences on the cAMP signaling including Rmax response. Nevertheless, we could not in a clear way suggest the correlation between cAMP signaling and phospho-ERK1/2 in the presented study. Although this study did not fully explore the relationship, the highly conserved regions, such as the trileucine and palmitoylation motifs in the C-terminus of eLH/CGR, likely participate in specific signaling pathways, such as receptor internalization. Therefore, the conserved regions in C-terminal of LH/CGRs should be systematically identified to which signals are pathway. We propose that the trileucine motif plays a role in downstream signaling, possibly through cooperative interactions with other motifs.

In summary, phospho-ERK1/2 activation through LH/CGR-mediated signaling warrants further investigation to determine whether an alternative pathway involving downstream phosphorylation of MAPK pathway effectors exists.

## 5. Conclusions

Our study demonstrates that mutations in the trileucine motif of the C-terminus of eLH/CGR do not result in significant reductions in cAMP response, surface expression, receptor loss, or phospho-ERK1/2 activation. However, the cAMP signaling (EC_50_ value) in the eLH/CGR-AAA mutant decreased by two-fold, and the maximal responsiveness was 34% lower than in eLH/CGR-wt. Similar phospho-ERK1/2 activation patterns were observed across all mutants, with a sharp peak at 5 min followed by a rapid decline. The phospho-ERK1/2 levels did not differ significantly between the eLH/CGR-wt and eLH/CGR-AAA mutants. These findings suggest that there is no direct correlation between cAMP response and phospho-ERK1/2 activation. Further research on glycoprotein hormone receptors is needed to elucidate the mechanisms underlying agonist-receptor complex-mediated downstream signaling.

## Figures and Tables

**Table 1:** Bioactivity of equine LH/CGR mutants.

eLH/CG Receptors	cAMP Responses		
	Basal *^a^* (nM/10^4^ cells)	Log (EC_50_) (ng/mL)	Rmax *^b^* (nM/10^4^ Cells)
eLH/CGR-WT	1.2 ± 0.4	18.5 (15.4 to 13.1) *^c^*	233.9 ± 4.9
eLH/CGR-ALL	1.3 ± 0.9	4.6 ** (4.2 to 5.1)	236.1 ± 2.1
eLH/CGR-LAL	1.6 ± 1.1	14.3 (12.4 to 16.7)	164.4 ± 2.5 *
eLH/CGR-LLA	1.6 ± 1.7	19.7 (16.7 to 22.5)	161.9 ± 2.6 *
eLH/CGR-AAA	1.7 ± 1.3	35.2 ** (31.1 to 40.9)	154.3 ± 2.4 *

Values are the means ± standard error of the mean from triplicate experiments. The log (EC_50_) values were determined from the concentration-response curves obtained from the in vitro bioassays. *^a^* Basal cAMP level average without agonist treatment. *^b^* Rmax average cAMP level/10^4^ cells. *^c^* Geometric means (95% confidence limit) of at least three experiments. *, ** Values with different superscripts are significantly different (*p* < 0.05, *p* < 0.01).

**Table 2:** Rates of cell surface loss of receptors in transiently transfected cell lines expressing the wild-type equine LH/CGR and mutants thereof.

eLH/CGR Cell Lines	t1/2 (min)	Plateau (% of Control)
eLH/CGR-WT	6.3 ± 0.2 *	74.9 ± 0.6
eLH/CGR-ALL	5.7 ± 0.1 *	81.2 ± 1.6
eLH/CGR-LAL	8.0 ± 0.3 *	77.5 ± 0.6
eLH/CGR-LLA	5.4 ± 0.2 *	71.1 ± 0.6
eLH/CGR-AAA	3.1 ± 0.1 **	78.9 ± 0.3

The data were fitted to one-phase exponential decay curves to obtain the values of t1/2 and the plateau (i.e., maximum reduction). Data were obtained from three independent experiments. *, ** Values with different superscripts are significantly different (*p* < 0.05).

## Data Availability

Data contained within the article.

## References

[1] Lefkowitz R.J., Shenoy S.K. (2005). Tranduction of receptor signals by b-arrestins. Science.

[2] Lefkowitz R.J., Rockman H., Shim P.J., Liu S., Ahn S., Pani B., Rajagopai S., Shenoy S.K., Bouvier M., Benovic J.L. (2023). How carvedilol does not activate b2-adrenoceptors. Nat. Commun..

[3] Choi S.H., Byambaragchaa M., Kim D.J., Lee J.H., Kang M.H., Min K.S. (2023). Specific signal transduction of constitutively activating (D576G) and inactivating (R476H) mutants of agonist-stimulated luteinizing hormone receptor in eel. Int. J. Mol. Sci..

[4] Min K.S., Park J.J., Byambaragchaa M., Kang M.H. (2019). Characterization of tethered equine chorionic gonadotropin and its deglycosylated mutants by ovulation stimulation in mice. BMC Biotechnol..

[5] Min K.S., Park J.J., Lee S.Y., Byambragchaa M., Kang M.H. (2020). Comparative gene expression profiling of mouse ovaries upon stimulation with natural equine chorionic gonadotropin (N-eCG) and tethered recombinant-eCG (R-eCG). BMC Biotechnol..

[6] Tao Y.X., Abell A.N., Liu X., Nakamura K., Segaloff D.L. (2000). Constitutive activation of G protein-coupled receptors as a result of selective substitution of a conserved leucine residue in transmembrane helix III. Mol. Endocrinol..

[7] Bhaskaran R.S., Ascoli M. (2005). The post-endocytotic fate of the gonadotropin receptors is an important determinant of the desensitization of gonadotropin responses. J. Mol. Endocrinol..

[8] Martinelle N., Holst M., Soder O., Svechnikov K. (2004). Extracellular signal-regulated kinases are involved in the acute activation of steroidogenesis in immature rat Leydig cells by human chorionic gonadotropin. Endocrinology.

[9] Tao Y.X. (2006). Inactivation mutations of G protein-couped receptors and disease: Structure-function insights and therapeutic implications. Pharmacol. Ther..

[10] Shiraishi K., Ascoli M. (2007). Lutropin/choriogonadotropin stimulate the proliferation of primary cultures of rat Leydig cells through a pathway that involves activation of the extracellularly regulated kinase 1/2 cascade. Endocrinology.

[11] Kim J.M., Byambaragchaa M., Kang M.H., Min K.S. (2018). The C-terminal phosphorylation sites of eel follicle-stimulating hormone receptors are important role in the signal transduction. Dev. Reprod..

[12] Min K.S., Liu X., Fabritz J., Jaquette J., Abell A.N., Ascoli M. (1988). Mutations that induce constitutive activation and mutations signal transduction modulate the basal and/or agonist-stimulated internalization of the lutropin/choriogonadotropin receptor. J. Biol. Chem..

[13] Ascoli M. (2007). Potential Leydig cell mitogenic signals generated by the wild-type and constitutively active mutants of the lutropin/choriogonadotropin receptor (LHR). Mol. Cell. Endocrinol..

[14] Jones B., McGlone E.R., Fang Z., Pickford P., Correa I.R., Oishi A., Jockers R., Inoue A., Kumar S., Gorlitz F. (2021). Genetic and biased agonist-mediated reductions in β-arrestin recruitment prolong cAMP signaling at glucagon family receptors. J. Biol. Chem..

[15] Shenoy S.K., Barak L.S., Xiao K., Ahn S., Berthouze M., Shukla A.K., Luttrell L.M., Lefkowitz R.J. (2007). Ubiquitination of β-arrestin links seven-transmembrane receptor endocytosis and ERK activation. J. Biol. Chem..

[16] Shenoy S.K., Draka M.T., Nelson C.D., Houtz D.A., Xiao K., Madabushi S., Reiter E., Premont R.T., Lichtarge O., Lefkowitz R.J. (2006). β-arrestin-dependent, G protein-independent ERK1/2 activation by the β2 adrenergic receptor. J. Biol. Chem..

[17] Slosky L.M., Bai Y., Toth K., Ray C., Rochelle L.K., Badea A., Chandrasekhar R., Pogorelov V.M., Abraham D.M., Atluri N. (2020). β-arrestin-biased allosteric modulated of NTSR1 selectively attenuates addictive behaviors. Cell.

[18] Kara E., Crepieux P., Gauthier C., Martinat N., Piketty V., Guillou F., Reiter E. (2006). A phosphorylation cluster of five serine and threonine residues in the C-terminus of the follicle-stimulating hormone receptor is important for desensitization but not for β-arrestin-mediated ERK activation. Mol. Endocrinol..

[19] Piketty V., Kara E., Guillou F., Reiter E., Crepiux P. (2006). Follicle-stimulating hormone (FSH) activates extracellular signal-regulated kinase phosphorylation independently of beta-arrestin- and dynamin-mediated FSH receptor internalization. Reprod. Biol. Endocrinol..

[20] Luttrell L.M., Wang J., Plouffe B., Smith J.S., Yamani L., Kaur S., Jean-Charles P.Y., Gauthier C., Lee M.H., Pani B. (2018). Manifold roles of β-arrestin in GPCR signaling elucidated with siRNA and CRISPR/Cas9. Sci. Signal..

[21] Shiraishi K., Ascoli M. (2006). Activation of the lutropin/choriogonadotropin receptor (LHR) in MA-10 cells stimulates tyrosine kinase cascade that activates Ras and the extracellular signal regulated kinases (ERK1/2). Endocrinology.

[22] Banerjee A.A., Dupakuntla M., Pathak B.R., Mahale S.D. (2015). FSH receptor-specific residues L^501^ and I^505^ in extracellular loop 2 are essential for its function. J. Mol. Endocrinol..

[23] Banerjee A.A., Mahale S.D. (2018). Extracellular loop 3 substitutions K589N and A590S in FSH receptor increase FSH-induced receptor internalization and along with S588T substitution exhibit impaired ERK1/2 phosphorylation. Arch. Biochem. Biophys..

[24] Faure M., Voyno-Yasenetskaya T.A., Bourne H.R. (1994). cAMP and beta gamma subunits of heterotrimeric G protein stimulate the mitogen-activated protein kinase pathway n COS-7 cells. J. Biol. Chem..

[25] Rodriguez M.C., Mussio P.E., Villarraza J., Tardivo M.B., Antuna S., Fontana D., Ceaglio N., Prieto C. (2023). Physiochemical characterization of a recombinant eCG and comparative studies with PMSG commercial preparations. Protein J..

[26] Sherman G.B., Wolfe M.W., Farmerie T.A., Clay C.M., Threadgill D.S., Sharp D.C., Nilson J.H. (1992). A single gene encodes the β-subunits of equine luteinizing hormone and chorionic gonadotropin. Mol. Endocrinol..

[27] Min K.S., Hattori N., Aikawa J.I., Shiota K., Ogawa T. (1996). Site-directed mutagenesis of recombinant equine chorionic gonadotropin/luteinizing hormone: Differential role of oligosaccharides in luteinizing hormone- and follicle-stimulating hormone-like activities. Endocrine J..

[28] Min K.S., Hiyama T., Seong H.W., Hattori N., Tanaka S., Shiota K. (2004). Biological activities of tethered equine chorionic gonadotropin (eCG) and its deglycosylated mutants. J. Reprod. Dev..

[29] Nakamura K., Ascoli M. (1999). A dileucine-based motif in the C-terminal tail of the lutropin/choriogonadotropin receptor inhibits endocytosis of the agonist-receptor complex. Mol. Pharmacol..

[30] Byambaragchaa M., Seong H.K., Choi S.H., Kim D.J., Kang M.H., Min K.S. (2021). Constitutively activating mutants of equine LH/CGR constitutively induce signal transduction and inactivating mutations impair biological activity and cell-surface receptor loss in vitro. Int. J. Mol. Sci..

[31] Gabilondo A.E., Hegler J., Krasel C., Boivin-Jahns V., Hein L., Lohse M.J. (1997). A dileucine motif in the C terminus of the β_2_-adrenergic receptor is involved in receptor internalization. Proc. Natl. Acad. Sci. USA.

[32] Zhu H., Wang H., Ascoli M. (1995). The lutropin/choriogonadotropin receptor is palmitoylated at intracellular cysteine residues. Mol. Endocrinol..

[33] Petaja-Repo U.E., Hogue M., Leskela T.T., Markkanen P.M.H., Tuusa J.T., Bouvier M. (2006). Distinct subcellular localization for constitutive and agonist-modulated palmitoylation of the human δ opioid receptor. J. Biol. Chem..

[34] Storch S., Phhl S., Braulke T. (2004). A dileucine motif and a cluster of acidic amino acids in the second cytoplasmic domain of the battern disease-related CLN2 protein are required for efficient lysosomal targeting. J. Biol. Chem..

[35] Jean-Charles P., Kaur S., Shenoy S.K. (2017). GPCR signaling via β-arrestin-dependent mechanisms. J. Cardiovasc. Pharmacol..

[36] Moritz A.E., Madaras N.S., Rankin M.L., Inbody L.R., Sibley D.R. (2023). Delineation of G protein-coupled receptor kinase phosphorylation sites within the D (1) dopamine receptor and their roles in modulating beta-arrestin binding and activation. Int. J. Mol. Sci..

[37] Guillien M., Mouhand A., Sagar A., Fournet A., Allemand F., Pereira G.A.N., Thureau A., Bernado P., Baneres J., Sibille N. (2023). Phosphorylation motif dictates GPCR c-terminal domain conformation and arrestin interaction. Structure.

[38] Byambaragchaa M., Park H.K., Kim D.J., Lee J.H., Kang M.H., Min K.S. (2022). The N-linked glycosylation site N191 is necessary for PKA signal transduction in eel follicle-stimulating hormone receptor. Int. J. Mol. Sci..

[39] Byambaragchaa M., Kim J.S., Park H.K., Kim D.J., Hong S.M., Kang M.H., Min K.S. (2020). Constitutive activation and inactivation of mutations inducing cell surface loss of receptor and impairing of signal transduction of agonist-stimulated eel follicle-stimulating hormone receptors. Int. J. Mol. Sci..

[40] Reiter E., Lefkowitz R.J. (2006). GRKs and β-arrestins: Roles in receptor silencing, trafficking and signaling. Trends Endocrinol. Metab..

[41] Shiraishi K., Ascoli M. (2008). A co-coculture system reveals the involvement of intercellular pathways as mediators of the lutropin receptor (LHR)-stimulated ERK1/2 phosphorylation in Leydig cells. Exp. Cell Res..

[42] Moller T.C., Pedersen M.F., van Senten J.R., Seiersen S.D., Mathiesen J.M., Bouvier M., Brauner-Osborne H. (2020). Dissecting the roles of GRK2 and GRK3 in mu-opioid receptor internalization and β-arrestin2 recruitment using CRISPR/Cas9-edited HEK293 cells. Sci. Rep..

[43] Ahn S., Shenoy K.K., Wei H., Lefkowitz R.J. (2004). Differential kinetic and spatial patterns of β-arrestin and G protein mediated ERK activation by the angiotensin II receptor. J. Biol. Chem..

[44] Hirakawa T., Galet C., Ascoli M. (2002). MA-10 cells transfected with the human lutropin/choriogonadotropin receptor (hLHR): A novel experimental paradigm to study the functional properties of the hLHR. Endocrinology.

